# Ultrasound-Guided Versus Traditional Refill of Intrathecal Infusion Pumps: A Prospective Quasi-Experimental Clinical Study

**DOI:** 10.3390/biomedicines13112671

**Published:** 2025-10-30

**Authors:** Beatriz Lechuga Carrasco, Jose Manuel Martínez-Linares, José Alejandro Ávila-Cabreja, Jonathan Cortés-Martín, Beatriz Piqueras-Sola, Nicolás Cordero Tous, Rafael Gálvez Mateos

**Affiliations:** 1 Virgen de las Nieves University Hospital, 18014 Granada, Spain; beatrizlcarrasco@gmail.com (B.L.C.); bpiquerassola@gmail.com (B.P.-S.); rafaelgalvez@hotmail.com (R.G.M.); 2 Department of Nursing, Faculty of Health Sciences, University of Granada, 18012 Granada, Spain; jmmartinezl@ugr.es; 3 Department of Research Methodology and Statistics, Fundación Pública Andaluza para la Investigación Biosanitaria Andalucía Oriental, 18012 Granada, Spain; javila@fibao.es

**Keywords:** intrathecal infusion pump, pain, re-loading portal, time, ultrasound

## Abstract

**Background:** There are multiple treatments and approaches available to manage pain. However, when these interventions fail to achieve adequate relief, pain management may involve the implantation of intrathecal infusion pumps. The use of ultrasound guidance may enhance nursing practice by improving procedural efficiency and patient comfort. This technique offers a more precise, safer, and less painful approach, potentially increasing patient satisfaction and reducing procedural complications. **Aim:** To evaluate patient pain levels during intrathecal infusion pump refills under ultrasound guidance compared to the traditional approach, also aiming to determine the time taken per technique and to assess related intra-procedural complications. **Design:** A prospective quasi-experimental pre-post study. **Methods:** The study population included individuals with intrathecal infusion pumps. Each participant underwent two refill visits: one using the traditional method and the subsequent refill using ultrasound guidance. The time required to complete the procedure, any complications, and pain related to the procedure were measured for both techniques. Time was measured using a stopwatch, and pain was assessed at the end of each procedure using a Visual Analogue Scale (VAS). **Results:** A total of 45 patients (25 men and 20 women), with a median age of 56.0 years were included. The estimated mean refill duration was 13.67 min for the traditional method versus 7.26 min for ultrasound-guided refills, representing a 53.2% reduction. The adjusted mean VAS was 2.76 (2.27–3.24) with ultrasound versus 5.91 (5.20–6.62) with the traditional method, yielding an adjusted mean difference of −3.16 (−4.02 to −2.30; *p* < 0.001). Reductions were consistent across subgroups defined by sex, refill duration, inter-procedural interval, intrathecal medication, and medical history. Complications occurred in 20.0% of traditional refills but in none of the ultrasound-guided procedures. **Conclusions:** Ultrasound guidance significantly reduces pain, complications, and procedure time, positioning it as the new standard of care for intrathecal pump refills. This mandates its immediate integration into nursing protocols and health management policies.

## 1. Introduction

Pain is defined as “an unpleasant sensory and emotional experience associated with, or resembling that associated with, actual or potential tissue damage” [1]. This definition highlights its subjective nature, with individual perception shaped by biological, psychological, and social factors. The nursing profession plays a fundamental role in the assessment, management, and ongoing care of patients experiencing pain. Nurses are essential in optimizing therapeutic strategies, ensuring patient comfort, and promoting adherence to complex treatment regimens, especially in chronic pain settings [2].

Chronic pain is a prevalent global health issue, affecting an estimated 20% of the adult population and significantly impacting quality of life, emotional well-being, and functional independence [3]. It is a complex, multifactorial condition that can be classified into various types, including nociceptive, neuropathic, and mixed pain. When pain becomes persistent and refractory to conventional treatments, it often requires more advanced interventions, such as intrathecal drug delivery systems [4].

For patients with severe, treatment-resistant pain, the implantation of intrathecal infusion pumps offers a targeted and effective method of medication delivery. These systems allow for continuous administration of analgesic drugs directly into the intrathecal space, improving symptom control while minimizing systemic side effects [5]. As such, they are a cornerstone of interventional pain management for select patient populations.

### 1.1. Background

There are multiple treatments and approaches available to manage pain. However, when these interventions fail to achieve adequate relief, pain management may involve the implantation of intrathecal infusion pumps [6]. This option is typically considered for patients with chronic pain in whom other procedures or pharmacological therapies have been ineffective.

These devices contain a reservoir, usually implanted in the abdominal area, into which medication is introduced for continuous administration to control pain. The refill process is performed via a portal, usually at intervals of 1 to 3 months depending on the medication type and prescribed dosage [7]. The refill requires a percutaneous puncture to access the portal. The portal exhibits ultrasound characteristics that facilitate its visualization [8,9]. This puncture is often performed “blindly” or using a plastic template. However, several studies have shown that ultrasound can be used to locate the refill port, especially in difficult cases [10,11,12,13,14,15,16,17], which may help reduce pain during the procedure [14,17,18], prevent complications [9,10,12,13,14,15,16,19], and make the refill process easier and safer [9,10,11,12,14,15,16,17,19,20]. Regarding the time required for the refill procedure, Stone [17] conducted a study involving 17 patients and found no statistically significant differences in the time spent between the ultrasound-guided group and the traditional method group.

### 1.2. The Study

This study originated at the Virgen de las Nieves Hospital in Granada, driven by the need to improve the approach to patients suffering from pain. Reviewing the published literature on the subject, it was observed that there is an evident improvement in the management of these patients thanks to the implementation of ultrasound guidance. The change is being implemented gradually and based on the results of this study.

The nurse who performed the procedures in this study was always the same and was already familiar with the use of the ultrasound device. For this specific protocol, she received standardized training lasting 90 min. The learning curve was exponential, excellent, and rapid, improving as the study progressed. Although a formal analysis of the curve was not conducted, the observed reduction in time suggests that the additional experience contributed to optimizing the procedure.

The implementation of ultrasound guidance did not generate any additional cost, as the service already possessed the ultrasound device. Therefore, the profitability analysis focuses on avoided indirect costs. The precise quantification of costs associated with avoided complications will be addressed in the forthcoming economic modeling phases of the project.

Finally, while a formal survey was not conducted, immediate verbal feedback was obtained from patients during the procedure. The expressions of relief and the perception of a faster and more comfortable process by the patients correlated with the quantitative results of pain reduction (VAS) and time. This initial qualitative feedback will be formalized in a satisfaction survey in the next stage of the research.

The primary objective of this study was to evaluate patient pain levels during intrathecal infusion pump refills under ultrasound guidance compared to the traditional approach, also aiming to determine the time taken per technique and to assess related intra-procedural complications.

## 2. Methods

### 2.1. Design

This was a quasi-experimental pre-post study involving individuals with intrathecal infusion pumps, in whom the refill procedure was performed using template guidance and ultrasound guidance in two different visits. The STROBE (STrengthening the Reporting of OBservational studies in Epidemiology) guidelines for observational studies were followed [21].

### 2.2. Study Setting and Sampling

The study was conducted at the Pain Unit of Hospital Virgen de las Nieves in Granada, Spain. Patients with intrathecal infusion devices who attended the unit to receive medication refills were included in the study. Refill visits usually occur every 1 to 3 months, depending on the dosage and type of medication prescribed.

A total of 45 patients with fixed-flow intrathecal infusion systems who agreed to participate in the study and signed the informed consent form were selected. At the time of study initiation, the Pain Unit had a total of 78 patients with continuous-flow intrathecal pumps, making the selected sample size representative for reliable data collection.

Each participant underwent two refill visits: one using the traditional method and the subsequent refill using ultrasound guidance.

The key influencing factor considered in the Visual Analogue Scale (VAS) analysis was the port localization strategy: traditional method versus ultrasound guidance.

Given the intra-patient paired design employed, each subject acted as their own control, which allowed the analysis to focus on the mean pain difference generated by the variation in technique. Indeed, a statistically significant variation was observed between both methods: the VAS scores reported during ultrasound-guided refill were consistently lower compared to the traditional approach, confirming the superiority of the ultrasound-guided method in reducing procedural pain in all patients. These data are presented in more detail in the results section.

### 2.3. Inclusion/Exclusion Criteria

Patients with intrathecal infusion pumps, adult (≥18 years old) were selected for the study. Only patients with continuous flow infusion devices were included, as these were equipped with a refill kit that included the template used in the “traditional method”.

Inclusion and exclusion criteria were strictly applied during patient selection. Participants received an information sheet explaining the procedure, along with an informed consent form authorizing the use of their data for research purposes. To ensure anonymity, each patient was assigned a unique study identification number, without collecting any personally identifiable information. These measures enhanced the validity and reliability of the data collected.

### 2.4. Study Interventions

#### 2.4.1. Traditional Method

For the traditional method, the sterile template provided by the manufacturer was used to refill the intrathecal infusion pumps. The pump was first located by manual palpation. After donning sterile gloves, the template was placed on the skin to identify the refill port of the infusion pump. The template remained in place throughout the refill procedure. The refill was performed through the template’s designated opening, which is positioned directly over the refill port [16].

#### 2.4.2. Ultrasound-Guided Method

For the ultrasound-guided method, the refill port was located using an ultrasound device. As the port is made of silicone, it appears as a vertically anechoic area on ultrasound imaging. In contrast, the pump’s metallic structure appears hyperechoic. Surrounding tissues, such as muscle and connective fibers, generate few returning echoes and thus appear hypoechoic. After locating the pump by palpation, the refill port was marked on the skin using a skin marker under ultrasound guidance. In more complex cases, such as when the device is not well anchored to the surrounding tissue, ultrasound can be used in real-time during the refill procedure with a sterile probe cover. In this study, ultrasound guidance during the actual refill was only required in one case, where the traditional method was not feasible [10].

### 2.5. Data Collection

Refills using the traditional method were performed by randomly selecting patients when their refill date was due. For ultrasound-guided refills, patients who were already scheduled to be reloaded by the same clinician were selected, ensuring consistency in operator technique. All patients were scheduled for their next refill based on the standard interval, typically between one and three months later.

Study variables were carefully planned in advance for appropriate methodological analysis, and recorded in a structured database. Data collected were sociodemographic variables including sex and age, type of intrathecal medication used (morphine vs. ziconotide), procedure time for each technique, complications during the procedure, and pain levels reported for each refill (traditional method vs. ultrasound-guided).

Time was measured using a stopwatch, started when the patient was lying on the examination table and ready to begin, and stopped once the needle was withdrawn. Pain level was assessed at the end of each procedure using the Visual Analogue Scale (VAS) [22] where participants rated their pain related to the refill process, in a scale ranging from 0 to 10 (0 = no pain, and 10 = the worst possible pain). The Visual Analogue Scale (VAS) is a standardized, unidimensional psychometric instrument for the subjective quantification of pain intensity. In its 0 to 10 format, it is defined by two polar anchors: 0: Represents no pain (absence of pain). 10: Denotes the worst imaginable pain (maximum intensity).

Complications during the procedure included multiple punctures, difficulty locating the reloading portal, and the need to use ultrasound or request assistance from another professional. The complications were not predefined and were recorded as they appeared.

It is important to note that the Visual Analogue Scale (VAS) was administered immediately upon completion of each intervention, without a predetermined or variable waiting period for individual patients. Pain was assessed for all patients at the conclusion of the procedure. Pain assessment was self-reported by the patients, and the operators were aware of the study.

Concurrently, to observe potential complications arising from each intervention, the observation period precisely matched the duration of the procedure in each case. Given the nature of the potential complications examined in this report, extending the observation period further was deemed unnecessary.

### 2.6. Data Analysis

We prespecified a superiority sample size for the paired VAS difference, assuming an expected mean difference of 0.5 points, a superiority margin of 1.0 point, a within-patient change standard deviation of 1.5, a type 1 error of 0.05, and 90% power. The minimum required sample size was 39 participants. In the absence of comparable studies [23] all planning parameters were investigator-defined.

The primary endpoint was procedural pain on a 0–10 VAS. We fitted a generalized linear mixed-effects model with a Poisson family and log link, including a patient-level random intercept to account for within-patient correlation. Fixed effects comprised technique (ultrasound vs. traditional), sex, inter-procedural interval (days), refill duration (minutes), intrathecal medication, and medical history categories. Model estimates were presented as adjusted marginal means with 95% confidence intervals for each technique and as the adjusted mean difference (ultrasound minus traditional) with 95% confidence intervals and two-sided *p* values. Average marginal effects were computed from the fitted model, and results are displayed on the original VAS.

The secondary endpoint was procedure time. We modeled time with a lognormal mixed-effects specification including treatment as the primary fixed effect. We report back-transformed adjusted geometric means and the model-based absolute difference in minutes.

Statistical significance was defined at α = 0.05 (two-tailed), with no interim analyses performed. Model assumptions were verified through residual diagnostics and Q-Q plots.

### 2.7. Ethical Considerations

The study complied with the Declaration of Helsinki regarding research involving human subjects. Ethical approval was obtained from the Research Ethics Committee (CEIM/CEI Provincial de Granada) on 30 June 2022, under reference number PEIBA_SEG 6 PLANT22052710190, with the study title: “Importance of Ultrasound for Nursing in the Detection of the Refill Port in Intrathecal Analgesic Infusion Pumps”.

Informed consent was obtained from the participants before starting any study procedure.

Patient anonymity was guaranteed by assigning a unique identification number to each participant. No personal data that could identify individuals was collected. Additionally, all data were stored on a single password-protected computer.

## 3. Results

### 3.1. Patients’ Characteristics

The study enrolled 45 patients (25 male [55.6%], 20 female [44.4%]) with a median age of 56.0 years (IQR: 51.0–64.0; range: 33.0–82.0). Primary indications for intrathecal therapy included lumbar pain/spinal degenerative disease (35.6%, *n* = 16), postsurgical spinal pain (28.9%, *n* = 13), and chronic widespread musculoskeletal pain (24.4%, *n* = 11), while less frequent conditions comprised tumors/rare diseases (6.7%, *n* = 3), structural abnormalities (2.2%, *n* = 1), and other etiologies (2.2%, *n* = 1). Most patients received intrathecal morphine (84.4%, *n* = 38) versus ziconotide (15.6%, *n* = 7), with a median inter-procedural interval of 72.0 days (IQR: 49.0–84.0; range: 21.0–94.0) between refill techniques (Table 1).

### 3.2. Factors Associated with Procedural Pain and Complications During Intrathecal Refills

In the adjusted mixed-effects model, ultrasound guidance was independently associated with lower procedural pain compared with the traditional technique (β −0.52, 95% CI −0.88 to −0.17; *p* = 0.004). Male sex showed lower pain scores than female (β −0.33, 95% CI −0.56 to −0.09; *p* = 0.008). A longer interval between procedures was associated with a small reduction in pain (β −0.07 per day, 95% CI −0.01 to −0.00; *p* = 0.029). Refill duration demonstrated no clear association (β 0.04 per minute, 95% CI −0.01 to 0.08; *p* = 0.093). Intrathecal medication type and medical history categories were not significantly associated with VAS after adjustment. Full coefficient estimates and confidence intervals are presented in Supplementary Table S1.

The traditional method was associated with 9 complications (20.0%, 95% CI: 10.9–33.8%), while ultrasound-guided refills demonstrated no complications. Nine patients experienced complications: 3 patients required template relocation to locate the portal, 1 patient needed 2 punctures, 2 patients needed 2 punctures using ultrasound guidance in the 2nd puncture, and 3 patients needed the use of ultrasound to find the portal.

Table 2 shows that ultrasound guidance was associated with substantially lower procedural pain than the traditional technique. The adjusted mean VAS was 2.76 (2.27–3.24) with ultrasound versus 5.91 (5.20–6.62) with the traditional method, yielding an adjusted mean difference of −3.16 (−4.02 to −2.30; *p* < 0.001). Reductions were consistent across subgroups defined by sex, refill duration, inter-procedural interval, intrathecal medication, and medical history. The largest absolute reduction was observed in patients with structural musculoskeletal abnormalities (adjusted difference −4.29; *p* = 0.002).

### 3.3. Comparison of Refill Duration and Pain Between Both Methods

**Figure 1:**
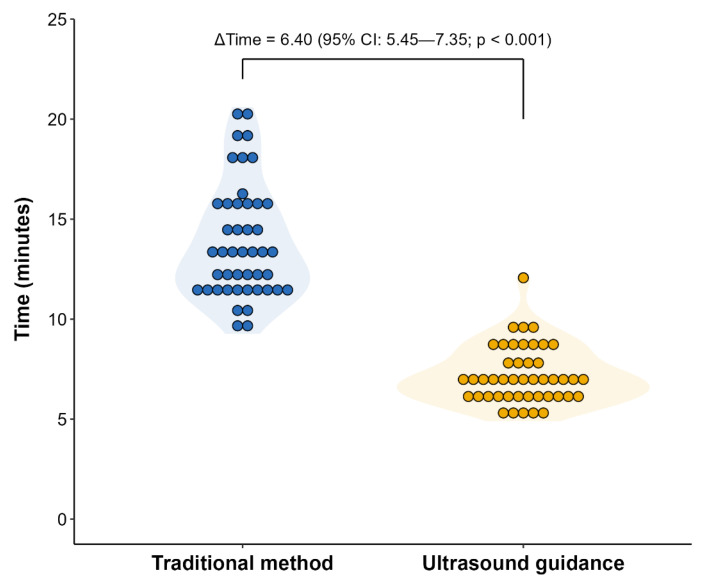
Comparison of refill procedure times between traditional and ultrasound-guided techniques.

The traditional method required significantly greater time to complete intrathecal pump refills compared to ultrasound guidance. Analysis via generalized linear mixed-effects modeling with lognormal distribution and subject-level random intercepts demonstrated a clinically substantial reduction in procedural duration with ultrasound guidance. The estimated mean refill time was 13.67 min (95% CI: 12.98–14.35) for traditional palpation versus 7.26 min (95% CI: 6.66–7.87) for ultrasound-guided refills. This represents a mean reduction of 6.40 min (95% CI: 5.63–7.18; *p* < 0.001), equivalent to a 53.2% decrease in refill duration with ultrasound guidance (Figure 1).

## 4. Discussion

The main objective of the present study was to evaluate patient pain levels during intrathecal infusion pump refills under ultrasound guidance compared to the traditional approach, also aiming to determine the time taken per technique and to assess related intra-procedural complications. Based on the results obtained and presented in the previous section, it can be observed that ultrasound guidance is a faster and less painful technique than the traditional template-guided method for refilling intrathecal infusion pumps. Specifically, ultrasound reduced the average procedure time by nearly half and significantly lowered patient-reported pain scores. These results are consistent with prior studies that highlight the advantages of ultrasound in improving procedural accuracy and comfort [9,14,15].

Although a previous randomized pilot study by Stone et al. [17] found no statistically significant difference in time between ultrasound and traditional methods, our study demonstrates a clear and clinically relevant reduction in procedure duration. This discrepancy may be attributable to differences in sample size, operator experience, or procedural setting. Additionally, Maino et al. [20] reported improved accuracy using ultrasound, although their focus was on refill success rates rather than procedure time or patient discomfort.

The reduction in pain observed in our study (VAS score reduction from 5.91 to 2.76) aligns with findings from Singa et al. [18], who reported increased patient comfort with ultrasound-guided refills. Pain during the refill process is often caused by multiple needle attempts or difficulty locating the port, issues that ultrasound can effectively mitigate [11,13]. In our study, the ultrasound method yielded a 100% success rate on the first attempt, compared to an 80% success rate with the traditional method, further supporting its reliability and precision.

The advantages of ultrasound guidance are not limited to time and pain reduction. By providing real-time visualization of the refill port, ultrasound enhances procedural safety and minimizes the risk of complications such as pocket fill or misplaced punctures [9,10,12]. This is particularly important in patients with challenging anatomy or in cases of pump displacement or fibrosis, where the refill port may be difficult to palpate [16].

These findings have profound implications, particularly for nursing practice and healthcare system efficiency [23]. Nurses, especially those in advanced practice roles or specializing in pain management, are central to the care of patients with implanted infusion devices. In many clinical settings, nurses bear the responsibility for conducting routine pump refills, monitoring for complications, managing refill schedules, and providing essential patient education [24,25]. The adoption of ultrasound-guided techniques empowers nurses to perform these refills with greater accuracy and patient comfort, significantly enhancing their procedural autonomy.

For high-volume pain units, integrating ultrasound is a powerful mechanism for system improvement [4]. This shift can substantially enhance workflow efficiency, reduce variability, and standardize outcomes across operators. It also creates opportunities for nurses to lead in both clinical practice and training. Recent evidence strongly supports this move toward nurse-led innovation, highlighting successful quality improvement initiatives led by nursing staff to standardize intrathecal care and minimize complications [26].

From a health systems perspective, the integration of ultrasound into refill procedures carries critical practical and policy implications [6]. The demonstrated shorter procedure times translate directly into increased clinic efficiency, the potential to reduce waiting lists, and optimized resource allocation. Moreover, the combination of increased first-attempt success rates and decreased patient discomfort strongly supports patient-centered care, which may reduce procedural anxiety and patient dropout rates. These improvements align perfectly with broader healthcare quality metrics and guidelines that prioritize safety and efficiency in interventional procedures.

This study offers immediate clinical findings while simultaneously laying the essential groundwork for future research aimed at a more holistic and profound understanding of this innovation. Having established the significant improvement in procedural time efficiency and reduction in patient pain perception, the next phase of evaluation must explore additional critical dimensions.

In this regard, it is imperative to quantify the economic impact of the technique by analyzing both short- and long-term operating costs. Likewise, future work must delve into patient safety outcomes, evaluating detailed metrics of adverse events. User satisfaction (from both the patient and the healthcare professional) constitutes another vital variable of interest to ensure the acceptance and sustainability of the method. Finally, research must extend to studying the long-term clinical benefits of ultrasound guidance on the quality of life and chronic pain management in this patient population.

These variables are recognized as essential pillars for an exhaustive evaluation and are already being incorporated and addressed in the forthcoming phases of this research project. Crucially, the execution of cost-effectiveness analyses is fundamental for informed decision-making in health policy, guiding the potential definitive integration of this technique into standardized national protocols for intrathecal therapy.

The study presents limitations inherent to its quasi-experimental design, specifically due to being non-randomized, single-center, and having a reduced sample size. The pre-post scheme used could have introduced a learning bias, given that the operator may have optimized their performance in the second (ultrasound-guided) refill thanks to previous experience with the patient. Although internal consistency was favored by using a single clinical team for all procedures, external validity is restricted. This suggests that the applicability of the results may be limited in other centers with different workflows, equipment, or staff expertise. There are also potential confounding factors such as anatomical variability.

Consequently, the findings of this work should be considered valuable hypothesis generators, particularly for high-volume pain units managing similar populations. We emphasize the critical need to conduct future randomized controlled trials (RCTs), with multicenter and larger samples that include diverse teams in order to confirm the transportability of the technique and strengthen the estimates of the effect found.

Despite these limitations, the study is supported by solid strengths: it was executed in a real-world clinical setting, relied on standardized data collection, and employed objective measures (VAS and stopwatch timing). The exhaustive inclusion of all eligible patients from a high-activity unit also ensures the representativeness of the sample. By documenting the feasibility and practical benefits of ultrasound guidance, this study provides fundamental evidence to drive innovation in interventional pain care, especially that led by nursing staff.

## 5. Conclusions

In conclusion, ultrasound guidance offers significant advantages over the traditional template method for refilling intrathecal infusion pumps. It is faster, less painful, and more reliable, with a higher success rate and fewer complications. These findings support the integration of ultrasound technology into routine nursing practice for managing intrathecal pump systems, particularly in chronic pain units. By improving both procedural efficiency and patient experience, ultrasound-guided refills represent a valuable innovation in the delivery of advanced pain management care. The performance of multicenter randomized controlled trials and cost-effectiveness studies is recommended to confirm and extend the current findings.

## Figures and Tables

**Table 1:** Sociodemographic and procedure-related characteristics.

Characteristic	*n* = 45 ^1^
Sex	
Female	20 (44.4%)
Male	25 (55.6%)
Age, years	
Min-Max	33.0–82.0
Mean (SD)	56.7 (10.9)
Median (Q1–Q3)	56.0 (51.0–64.0)
Medical history	
Postsurgical spinal pain	13 (28.9%)
Lumbar pain / spinal degenerative disease	16 (35.6%)
Chronic widespread musculoskeletal pain	11 (24.4%)
Structural musculoskeletal abnormalities	1 (2.2%)
Tumors / Rare diseases	3 (6.7%)
Other	1 (2.2%)
Intrathecal medication type	
Ziconotide	7 (15.6%)
Morphine	38 (84.4%)
Time between procedures, days	
Min-Max	21.0–94.0
Mean (SD)	64.7 (21.5)
Median (Q1–Q3)	72.0 (49.0–84.0)

^1^ *n* (%).

**Table 2:** Procedural pain by technique: adjusted VAS means for traditional vs. ultrasound guidance, with adjusted differences and subgroup results.

Variable ^1^	Traditional Method ^2^	Ultrasound Guidance ^2^	Adjusted Difference (95% CI) ^3^	*p*
Self-reported procedural pain	5.91 (5.20, 6.62)	2.76 (2.27, 3.24)	−3.16 (−4.02, −2.30)	<0.001
Stratified analysis				
Sex				
Female	6.74 (5.70, 7.79)	3.11 (2.48, 3.74)	−3.63 (−4.68, −2.59)	<0.001
Male	5.25 (4.42, 6.07)	2.47 (1.97, 2.97)	−2.77 (−3.59, −1.95)	<0.001
Refill duration, fixed at 10 min	5.12 (4.05, 6.20)	3.03 (2.40, 3.67)	−2.09 (−3.55, −0.63)	0.005
Time between procedures, fixed at 72 days	5.62 (4.89, 6.35)	2.61 (2.14, 3.09)	−3.01 (−3.84, −2.18)	<0.001
Intrathecal medication type				
Ziconotide	6.51 (4.89, 8.14)	3.06 (2.20, 3.91)	−3.46 (−4.67, −2.24)	<0.001
Morphine	5.80 (5.06, 6.54)	2.70 (2.21, 3.19)	−3.10 (−3.95, −2.24)	<0.001
Medical history				
Postsurgical spinal pain	5.77 (4.62, 6.92)	2.69 (2.06, 3.33)	−3.08 (−4.05, −2.11)	<0.001
Lumbar pain/spinal degenerative disease	5.90 (4.84, 6.97)	2.72 (2.12, 3.33)	−3.18 (−4.14, −2.23)	<0.001
Chronic widespread musculoskeletal pain	5.66 (4.44, 6.88)	2.70 (2.03, 3.38)	−2.95 (−3.95, −1.96)	<0.001
Structural musculoskeletal abnormalities	8.14 (3.50, 12.8)	3.86 (1.60, 6.11)	−4.29 (−6.96, −1.61)	0.002
Tumors/Rare diseases	6.69 (4.21, 9.17)	2.98 (1.80, 4.15)	−3.71 (−5.34, −2.09)	<0.001
Other	6.08 (2.08, 10.07)	2.92 (0.966, 4.88)	−3.15 (−5.38, −0.92)	0.006

^1^ For subgroup rows, estimates are subgroup-specific with other covariates held at their observed distribution unless otherwise specified. Where indicated, refill duration was fixed at 10 min and time between procedures at 72 days to aid comparability. ^2^ Predicted mean (95% CI) from a generalized linear mixed-effects model with Poisson family and log link, including a patient-level random intercept. Fixed effects comprised technique, sex, inter-procedural interval, refill duration, intrathecal medication, and medical history. Estimates are presented on the original 0–10 VAS; lower values indicate less pain. ^3^ Adjusted difference (95% CI) equals ultrasound guidance minus traditional method, obtained as the average marginal effect from the fitted model.

## Data Availability

Data regarding this study is available upon request to the corresponding author.

## Supplementary Materials

The following supporting information can be downloaded at: https://www.mdpi.com/article/10.3390/biomedicines13112671/s1, Supplementary Table S1. Fixed Effects Estimates from the Generalized Linear Mixed Model for Procedural Pain Scores (Visual Analog Scale).
