# Supplementary material for: Ultrasound-Guided Versus Traditional Refill of Intrathecal Infusion Pumps: A Prospective Quasi-Experimental Clinical Study
Source: Biomedicines. 2025 Oct 30;13(11):2671. doi: 10.3390/biomedicines13112671 (PMC12650629; doi:10.3390/biomedicines13112671)
Supplement: Supplementary file 1 [file biomedicines-13-02671-s001.zip › biomedicines-3874176-supplementary.pdf]

Supplementary Table S1. Fixed Effects Estimates from the Generalized Linear Mixed Model for Procedural Pain Scores (Visual Analog Scale)

**Characteristic**	**Beta**	**95% CI**	**p-value**
Intercept	1.484	0.669, 2.300	<0.001
Treatment			
Traditional method	—	—	
Ultrasound guidance	-0.524	-0.878, -0.170	0.004
Sex			
Female	—	—	
Male	-0.325	-0.563, -0.087	0.008
Refill duration, minutes	0.036	-0.006, 0.077	0.093
Time between procedures, days	-0.006	-0.012, -0.001	0.029
Treatment			
Ziconotide	—	—	
Morphine	-0.040	-0.403, 0.324	0.831
Medical history			
Postsurgical spinal pain	—	—	
Lumbar pain / spinal degenerative disease	-0.030	-0.292, 0.232	0.822
Chronic widespread musculoskeletal pain	-0.158	-0.458, 0.142	0.302
Structural musculoskeletal abnormalities	0.047	-0.569, 0.664	0.881
Tumors / Rare diseases	-0.122	-0.578, 0.334	0.601
Other	0.015	-0.736, 0.766	0.969

Abbreviation: CI, Confidence Interval
